# Significant increase in the prevalence of Panton–Valentine leukocidin-positive methicillin-resistant *Staphylococcus aureus*, particularly the USA300 variant ΨUSA300, in the Japanese community

**DOI:** 10.1128/spectrum.01248-23

**Published:** 2023-11-06

**Authors:** Hiroshi Kaneko, Miki Kanai, Takumi Saito, Yuka Yanagi, Hana Kobayashi, Rikuto Kurihara, Masami Ikeda, Osamu Nemoto, Naoko Baba, Yasushi Matsuzaki, Daisuke Sawamura, Fumiko Shimoe, Yoichi Inaba, Yoko Kobayashi, Satoru Kawasaki, Toru Ueki, Sakae Funatsu, Shigeho Shirahama, Misao Oba, Takaya Hasegawa, Hirotoshi Furukawa, Toshiko Miyata, Masaaki Isonokami, Shigeru Fujita, Hidemasa Nakaminami

**Affiliations:** 1 Department of Clinical Microbiology, School of Pharmacy, Tokyo University of Pharmacy and Life Sciences, Tokyo, Japan; 2 Department of Dermatology, Takamatsu Red Cross Hospital, Kagawa, Japan; 3 Sapporo Skin Clinic, Hokkaido, Japan; 4 Department of Dermatology, Kanagawa Children’s Medical Center, Kanagawa, Japan; 5 Department of Dermatology, Hirosaki University Graduate School of Medicine, Aomori, Japan; 6 Shimoe Dermatology Clinic, Osaka, Japan; 7 Atopia Clinic, Kumamoto, Japan; 8 Kobayashi Dermatology Clinic, Tokyo, Japan; 9 Kawasaki Dermatology Clinic, Tokyo, Japan; 10 Ueki Dermatology Plastic Surgery, Tokyo, Japan; 11 Kawano Dermatology Clinic, Tokyo, Japan; 12 Department of Dermatology, Seirei Mikatahara General Hospital, Shizuoka, Japan; 13 Hasegawa Dermatology Clinic, Fukushima, Japan; 14 Furukawa Dermatology Clinic, Fukushima, Japan; 15 Division of Dermatology, Saitama Citizens Medical Center, Saitama, Japan; 16 Isonokami Dermatological Clinic, Osaka, Japan; 17 Fujita Dermatological Clinic, Niigata, Japan; University of Calgary, Calgary, Alberta, Canada

**Keywords:** methicillin-resistant *Staphylococcus aureus*, Panton–Valentine leukocidin, USA300, ΨUSA300

## Abstract

USA300, a major Panton–Valentine leukocidin (PVL)-producing methicillin-resistant *Staphylococcus aureus* (MRSA) clone associated with skin and soft tissue infections (SSTIs), carries a type IV staphylococcal cassette chromosome *mec* (SCC*mec*) element. Recently, a USA300 variant, ΨUSA300, with a 12-bp deletion of *ccrB2* in the SCC*mec* element, was identified in Japan by Takadama et al. (15). To understand the current status of PVL-positive MRSA in the community, a nationwide surveillance of MRSA isolated from outpatients at dermatology departments was conducted. This study included 1,619 outpatients who visited dermatology departments at 22 healthcare facilities in 11 prefectures in Japan between 2018 and 2021; *S. aureus* were isolated from these outpatients. Molecular epidemiological analysis was performed using PCR, SCC*mec* and *spa* typing, pulsed-field gel electrophoresis, multilocus sequence typing, and antimicrobial susceptibility testing. A total of 980 *S*. *aureus* were isolated, 293 of which were identified as MRSA. Among MRSA isolates, PVL genes were detected in 18 of 63 (28.6%) in 2018, 34 of 85 (40.0%) in 2019, 29 of 65 (44.6%) in 2020, and 40 in 80 (50.0%) in 2021. Among the PVL-positive MRSA, 16 of 121 (13.2%) and 94 of 121 (77.7%) isolates were identified as USA300 and ΨUSA300, respectively. ΨUSA300 strains were collected from six prefectures in Japan: Kagawa, Osaka, Tokyo, Kanagawa, Fukushima, and Saitama. ΨUSA300 was isolated more frequently than USA300 from patients with deep-seated SSTIs (*P* < 0.05). Our findings showed that PVL-positive MRSA isolates were significantly increased in the Japanese community. Furthermore, the USA300 variant ΨUSA300 has emerged as the predominant PVL-positive MRSA clone across the Kanto, Kinki, and Shikoku regions of Japan, and this variant may possess a greater propensity to cause deep-seated SSTIs than in USA300.

**IMPORTANCE:**

USA300 is an MRSA clone producing PVL, a toxin associated with SSTIs. ΨUSA300 is a USA300 variant recently identified in Japan by Takadama et al. (15). Here, we found that the prevalence rate of PVL-positive MRSA in *S. aureus* was elevated in the Japanese community, and ΨUSA300 accounted for most of them. ΨUSA300 strains have been isolated from several areas in Japan and were associated with deep-seated SSTIs. This study highlighted the emerging threat posed by ΨUSA300 in Japan.

## INTRODUCTION

*Staphylococcus aureus* is a commensal bacterium that colonizes the nose and skin in approximately 30% of humans (1). Methicillin-resistant *Staphylococcus aureus* (MRSA), which is one of the most common antimicrobial-resistant bacteria, is known to cause serious diseases, such as bacteremia, endocarditis, and necrotizing pneumonia, mainly in healthcare settings (1). Meanwhile, in community settings, MRSA is a major cause of skin and soft tissue infections (SSTIs), such as impetigo, folliculitis, and furuncle (1, 2).

Panton–Valentine leukocidin (PVL) is a two-component toxin produced by *S. aureus* that exhibits cytotoxicity against human neutrophils via pore formation (3). PVL-positive *S. aureus* is frequently isolated from the community and is associated with deep-seated SSTIs, including furuncle, furunculosis, and abscess (3, 4). Additionally, it can cause fatal infections, including necrotizing pneumonia, in healthy individuals (4 – 6). PVL was identified in *S. aureus* in 1932 by Panton and Valentine (7). To date, diverse genotypes of PVL-positive MRSA have been reported worldwide (8, 9). USA300 is a sequence type (ST)8 epidemic clone carrying PVL genes, a type IV staphylococcal cassette chromosome *mec* (SCC*mec*) element, and a type I arginine catabolic mobile element (ACME), which have spread in the United States, Spain, Australia, Japan, and other countries (8, 10). Several variants of this clone have been reported (9, 10). USA300 has the ability to widely spread in community and healthcare settings (10, 11).

In Japan, PVL-positive MRSA was first isolated in 2003 (12), followed by USA300 in 2007 (13). USA300-LV/J and ΨUSA300 have also been identified in Japan (14, 15). The molecular characteristics of these clones are almost similar to those of USA300, with the following differences: USA300-LV/J lacks ACME and has acquired a copper and mercury resistance mobile element (COMER) (14), and ΨUSA300 carries a type IV SCC*mec* element with a 12-bp deletion in *ccrB2* (SCC*mec* type ΨIV) (15). ΨUSA300 was first identified as a USA300 variant that was misclassified as an isolate harboring SCC*mec* type I by conventional PCR-based SCC*mec* typing because of deletion in the binding site of a βc primer (15). The emergence of ΨUSA300 in Japan has been observed in previous studies (16 – 18).

In Japan, PVL-positive MRSA was first isolated in 2003 (12), followed by USA300 in 2007 (13). USA300-LV/J and ΨUSA300 have also been identified in Japan (14, 15). The molecular characteristics of these clones are almost similar to those of USA300, with the following differences: USA300-LV/J lacks ACME and has acquired a copper and mercury resistance mobile element (COMER) (14), and ΨUSA300 carries a type IV SCC*mec* element with a 12-bp deletion in *ccrB2* (SCC*mec* type ΨIV) (15). ΨUSA300 was first identified as a USA300 variant that was misclassified as an isolate harboring SCC*mec* type I by conventional PCR-based SCC*mec* typing because of deletion in the binding site of a βc primer (15). The emergence of ΨUSA300 in Japan has been observed in previous studies (16 – 18). The detection rate of PVL genes among MRSA has increased in community settings in many parts of Japan (17 – 20). Nationwide annual epidemiological data on MRSA in healthcare settings is limited. However, the prevalence of PVL genes among MRSA in hospitals across Japan is generally lower than that in clinics (21 – 26). PVL-positive MRSA tends to be isolated more frequently from the skin and soft tissue cultures than from blood cultures (24 – 26). We previously reported an increase in PVL-positive MRSA, primarily in the dermatology departments of hospitals in Tokyo (26). Most of the PVL-positive MRSA isolates from Japan in recent years have been classified as USA300 (17, 18, 24, 26). USA300 has recently emerged in Taiwan, South Korea, and neighboring countries of Japan (27, 28). Therefore, it is important to monitor the epidemiology of PVL-positive MRSA, particularly USA300 and its variants, in Japan. However, to date, only few nationwide epidemiological studies on MRSA have been conducted in the community. To understand the current status of PVL-positive MRSA in the Japanese community, a nationwide surveillance of MRSA isolated from outpatients at dermatology departments was conducted.

## MATERIALS AND METHODS

### Patients and bacterial strains

A total of 1,619 outpatients who visited the dermatology departments at 18 clinics and 4 hospitals in Japan between 2018 and 2021 were included in this study (Table S1). The patients were monitored by patient identifiers within each healthcare facility; isolates in the first culture of each patient were included during the study period. No exclusion criteria were applied. Among the 1,619 patients included in this study, 610 (37.7%), 591 (36.5%), and 327 (20.2%) were aged ≤14, 15–64, and ≥65 years, respectively (mean, 33.8 years; median, 28 years; range, 0–98 years). Of the 1,619 patients, age data were unavailable for 91 (5.6%) patients (Table S2). Out of the 1,619 patients, a total of 931 (57.5%) and 660 (40.8%) were male and female, respectively (male-to-female sex ratio, 1.41). Sex data were unavailable for 28 of the 1,619 (1.7%) patients. Based on the type of disease or lesion, sample sources were classified as superﬁcial SSTIs or deep-seated SSTIs or were deemed unclassifiable, based on previous reports (29, 30). Samples were obtained from routine diagnostics using regular rayon swabs with amies agar gel (Transystem 114C; COPAN Diagnostics Inc., California, USA). The swabs were streaked directly onto mannitol salt agar (Oxoid Ltd., Hampshire, UK). One or several propagated colonies were collected and confirmed to be Gram-positive cocci using Gram staining. This was followed by identification via coagulase production test using PS LATEX (Eiken Chemical Co., Ltd., Tokyo, Japan) and detection of *nuc* using PCR (17, 31). If both tests were positive, isolates were determined to be *S. aureus*; if either test was negative, bacterial species were determined using 16S ribosomal RNA gene sequences as previously reported (32). Only the isolates identified as *S. aureus* using these methods were used for subsequent analyses. MRSA isolates were conﬁrmed by the detection of *mecA* by PCR (17). All *S. aureus* isolates were obtained from different patients, except for those from whom both methicillin-susceptible *S. aureus* (MSSA) and MRSA were isolated (Table S1). The following *S. aureus* strains were used as SCC*mec*-type strains: NCTC10442 (type I), N315 (type II), 85/2082 (type III), JCSC4744 (type IV), TSI637 (type ΨIV), and WIS (type V) (15, 33). ATCC29213 was used as a quality control strain for antimicrobial susceptibility testing, and N315 and JCSC6774 were used as a DNA reference and a USA300 standard strain, respectively, for pulsed-field gel electrophoresis (PFGE) analysis (34, 35).

### PCR analysis, SCC*mec* and *spa* typing, PFGE analysis, and multilocus sequence typing

PCR analysis was performed for the presence of PVL genes (*lukS-PV* and *lukF-PV*), toxic shock syndrome toxin-1 (TSST-1) gene (*tst*), ACME genes (*arcA* and *opp-3C*), exfoliative toxin genes (*eta*, *etb*, and *etd*), staphylococcal enterotoxin genes (*sea*, *seb*, *sec*, *sed*, *see*, *seg*, *seh*, *sei*, and *sej*), and antimicrobial resistance genes [*mecA*, *aac(6′)-aph(2*″), *erm*(A), *erm*(C), *msr*(A/B), *mph*(C), *tet*(K), and *tet*(M)], as previously described (20, 36). SCC*mec* and *spa* typing, PFGE analysis, and multilocus sequence typing (MLST) were performed as previously described (17, 37). SCC*mec* type ΨIV was detected according to a previous study (15). The nucleotide sequences of *spa* and the housekeeping genes were determined using an Applied Biosystems 3500 Genetic Analyzer (Thermo Fisher Scientific Inc., Massachusetts, USA) and Phred (38). PFGE patterns were obtained by digestion of DNA with *Sma*I and analyzed using BioNumerics version 7.6 (Applied Maths, Sint-Martens-Latem, Belgium); a dendrogram of them was constructed using the unweighted pair group method with arithmetic mean using the Dice coefﬁcient with 1.0% optimization and 1.0% band position tolerance.

### Antimicrobial susceptibility testing

The minimum inhibitory concentrations (MICs) of the antimicrobial agents were determined using the agar dilution method according to the Clinical and Laboratory Standards Institute (CLSI) guidelines (M07-A11) (39). The following antimicrobial agents were used: ampicillin (Sigma-Aldrich Co., LLC, MO, USA), oxacillin (Tokyo Chemical Industry Co., Ltd., Tokyo, Japan), cefalexin (FUJIFILM Wako Pure Chemical Corporation, Osaka, Japan), cefdinir (Sigma-Aldrich), faropenem (Sigma-Aldrich), fosfomycin (Sigma-Aldrich), gentamicin (FUJIFILM Wako Pure Chemical Corporation), clarithromycin (Tokyo Chemical Industry), clindamycin (Tokyo Chemical Industry), levofloxacin (Tokyo Chemical Industry), nadifloxacin (Tokyo Chemical Industry), tetracycline (FUJIFILM Wako Pure Chemical Corporation), minocycline (Tokyo Chemical Industry), vancomycin (FUJIFILM Wako Pure Chemical Corporation), chloramphenicol (FUJIFILM Wako Pure Chemical Corporation), and fusidic acid (Tokyo Chemical Industry). The breakpoints for these antimicrobial agents were determined based on CLSI interpretation criteria (M100-S32) (40).

### Statistical analysis

Statistical analyses were performed using JMP Pro version 15 (SAS Institute Inc., North Carolina, USA). The number of patients and isolates was statistically compared using Fisher’s exact test. The age distribution of patients was statistically compared using Mann–Whitney U test. *P* values of <0.05 were considered statistically significant.

## RESULTS

### Identification and classiﬁcation of *S. aureus* isolates in the community

**Fig 1:**
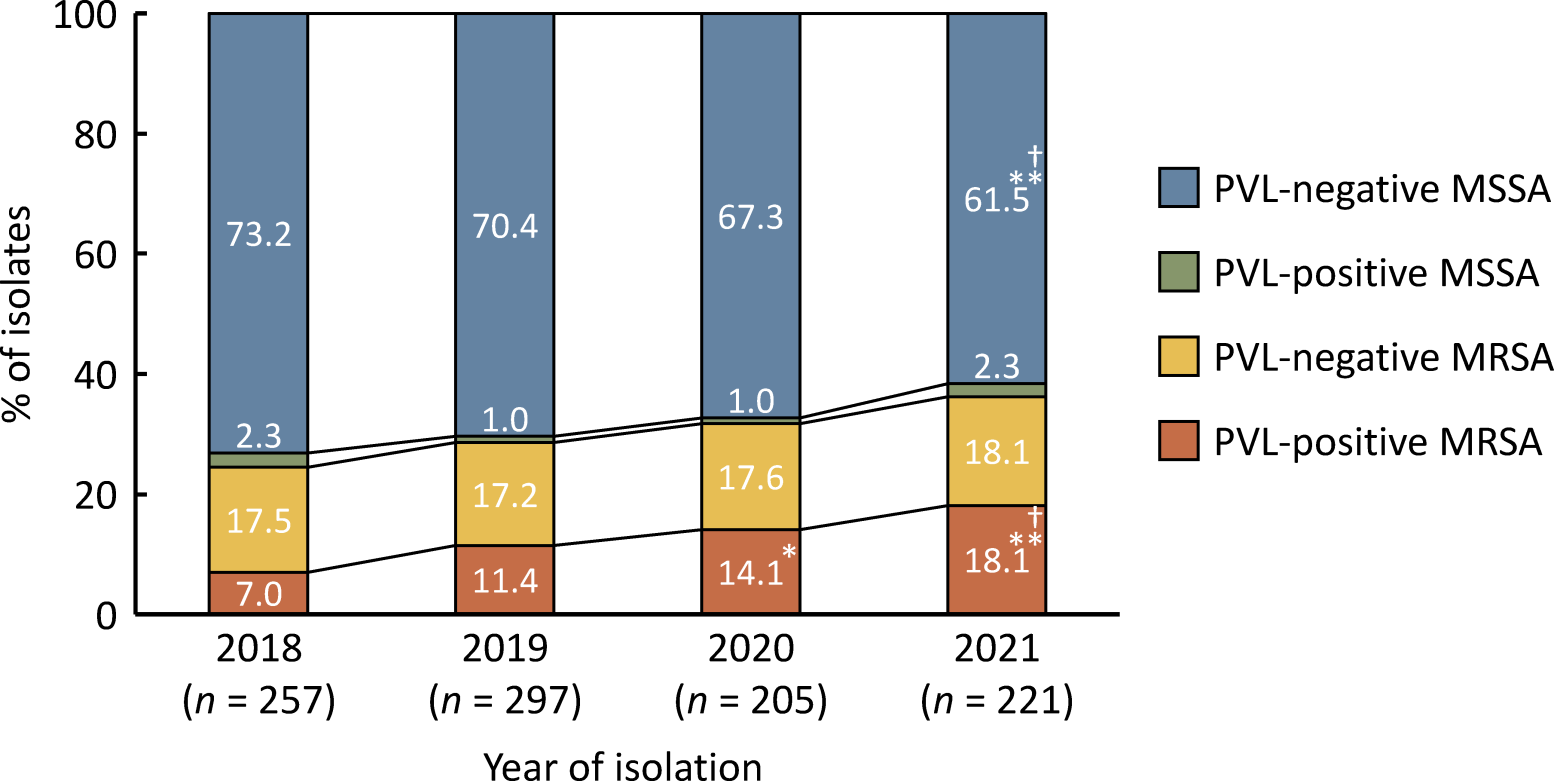
Annual proportions of PVL-positive and PVL-negative *S. aureus* isolates in Japan from 2018 to 2021. *P < 0.05, **P < 0.01 versus the percentage of isolates in 2018, and †*P* < 0.05 versus the percentage of isolates in 2019 by Fisher’s exact test.

A total of 980 *S*. *aureus* were isolated from 972 of 1,619 (60.0%) outpatients who had visited the dermatology departments (Table S1); both MSSA and MRSA were isolated from 8 patients. Among the 980 *S*. *aureus* isolates, 687 (70.1%) and 293 (29.9%) were identified as MSSA and MRSA, respectively. The numbers of MRSA isolates were 63 (24.5%) in 2018, 85 (28.6%) in 2019, 65 (31.7%) in 2020, and 80 (36.2%) in 2021 (Fig. 1). The ratio of MSSA to MRSA isolates in 2021 was significantly different from that in 2018 (*P* < 0.01).

PVL genes were detected in 16 (2.3%) and 121 (41.3%) of the MSSA and MRSA isolates, respectively. The numbers of PVL-positive MRSA isolates were 18 (28.6%) in 2018, 34 (40.0%) in 2019, 29 (44.6%) in 2020, and 40 (50.0%) in 2021 (Fig. 1). The ratio of PVL-positive to PVL-negative MRSA isolates in 2021 was significantly different from that in 2018 (*P* < 0.05). PVL-positive MRSA accounted for 3 of 5 (60.0%), 6 of 38 (15.8%), 20 of 52 (38.5%), 5 of 10 (50.0%), 11 of 14 (78.6%), and 76 of 159 (47.8%) of MRSA cases in the Hokkaido, Tohoku, Kanto, Chubu, Kinki, and Shikoku regions, respectively, whereas no PVL-positive MRSAs were observed in the Kyushu region (Fig. S1A). Significant differences in the detection rates of PVL genes among MRSA isolates were detected in certain regions. No significant differences in the detection rates of PVL genes in MRSA were observed between prefectures belonging to the same region, namely, Aomori and Fukushima; Saitama, Tokyo, and Kanagawa; and Niigata and Shizuoka (Table S3). The proportion of PVL-positive MRSA among *S. aureus* increased annually, whereas that of PVL-negative MSSA decreased during the study period (Fig. 1). The proportions of PVL-positive MRSA and PVL-negative MSSA significantly increased and decreased in 2021, respectively, compared to those in 2018 and 2019 (*P* < 0.01 and *P* < 0.05, respectively). There were no significant changes in the proportions of PVL-positive MSSA and PVL-negative MRSA among *S. aureus* spp.

The following types of SCC*mec* elements were detected in MRSA isolates: type I in 3 (1.0%), type II in 11 (3.8%), type IV in 100 (34.1%), type ΨIV in 94 (32.1%), type V in 76 (25.9%), and non-typeable in 9 (3.1%) isolates. No MRSA isolates with a type III SCC*mec* element were detected. All SCC*mec* type ΨIV isolates were positive for PVL genes.

### Epidemiological and clinical background of the patients

Of the 1,619 patients, a total of 622 (38.4%) and 562 (34.7%) were diagnosed with superﬁcial and deep-seated SSTIs, respectively, and 377 (23.3%) had unclassifiable diseases; the remaining patients did not have diagnostic data (Table S2). The age of the patients with superﬁcial SSTIs, primarily impetigo, was ≤14 years in 490 of 622 (78.8%) cases. Of the patients with deep-seated SSTIs, including furuncle, abscess, and paronychia, 298 of 562 (53.0%) patients reported ages between 15 and 64 years. The types of disease or lesion did not differ markedly between males and females.

*S. aureus* were isolated from 499 of 622 (80.2%) patients with superﬁcial SSTIs and 278 of 562 (49.5%) patients with deep-seated SSTIs (Table S2). PVL-positive isolates were detected more frequently than PVL-negative isolates in patients with deep-seated SSTIs (*P* < 0.01) (Table 1). There were no signiﬁcant diﬀerences in the prevalence rates of PVL-positive and PVL-negative MSSA versus MRSA in each classification of SSTIs. In addition, PVL-positive isolates tended to be isolated more frequently from 15–64 years of age than PVL-negative isolates (*P* < 0.01) (Table S4).

**TABLE 1:** Number (percentage) of *S. aureus* strains belonging to each genotype by classification of SSTIs^*e*^ ^,*f*^

Genotype of *S. aureus* strains	Superﬁcial SSTIs					Deep-seated SSTIs								Unclassifiable						ND
		Impetigo	Folliculitis	Pustule	Others		Furuncle	Furunculosis	Carbuncle	Abscess	Cellulitis	Paronychia	Others		Epidermal cyst	Hidradenitis suppurativa	Ulcer	Wound	Others	
PVL-negative MSSA (*n* = 671)	384 (57.2)	363 (54.1)	13 (1.9)	3 (0.4)	5 (0.7)	142 (21.2)	54 (8.0)	2 (0.3)	5 (0.7)	26 (3.9)	20 (3.0)	33 (4.9)	2 (0.3)	118 (17.6)	21 (3.1)	5 (0.7)	60 (8.9)	17 (2.5)	15 (2.2)	27 (4.0)
PVL-positive MSSA (*n* = 16)	0 (0.0)^*b*^	0 (0.0)^*b*^	0 (0.0)	0 (0.0)	0 (0.0)	11 (68.8)^*b*^	4 (25.0)^*a*^	2 (12.5)^*b*^	1 (6.3)	3 (18.8)^*a*^	0 (0.0)	1 (6.3)	0 (0.0)	5 (31.3)	3 (18.8)^*a*^	1 (6.3)	1 (6.3)	0 (0.0)	0 (0.0)	0 (0.0)
PVL-negative MRSA (*n* = 172)	101 (58.7)	97 (56.4)	3 (1.7)	0 (0.0)	1 (0.6)	27 (15.7)	6 (3.5)	1 (0.6)	1 (0.6)	6 (3.5)	3 (1.7)	10 (5.8)	0 (0.0)	36 (20.9)	2 (1.2)	4 (2.3)	20 (11.6)	5 (2.9)	5 (2.9)	8 (4.7)
PVL-positive MRSA (*n* = 121)	14 (11.6)^*b*^	6 (5.0)^*b*^	5 (4.1)	1 (0.8)	2 (1.7)	98 (81.0)^*b*^	42 (34.7)^*b*^	16 (13.2)^*b*^	6 (5.0)^*a*^	20 (16.5)^*b*^	11 (9.1)^*b*^	1 (0.8)^*a*^	2 (1.7)	6 (5.0)^*b*^	0 (0.0)	1 (0.8)	4 (3.3)^*b*^	1 (0.8)	0 (0.0)	3 (2.5)
USA300 (*n* = 16)	6 (37.5)	3 (18.8)	1 (6.3)	1 (6.3)	1 (6.3)	9 (56.3)	6 (37.5)	0 (0.0)	0 (0.0)	1 (6.3)	1 (6.3)	0 (0.0)	1 (6.3)	1 (6.3)	0 (0.0)	0 (0.0)	0 (0.0)	1 (6.3)	0 (0.0)	0 (0.0)
ΨUSA300 (*n* = 94)	8 (8.5)^*d*^	3 (3.2)^*c*^	4 (4.3)	0 (0.0)	1 (1.1)	80 (85.1)^*c*^	32 (34.0)	16 (17.0)	6 (6.4)	16 (17.0)	10 (10.6)	0 (0.0)	0 (0.0)	3 (3.2)	0 (0.0)	1 (1.1)	2 (2.1)	0 (0.0)	0 (0.0)	3 (3.2)
Others (*n* = 11)	0 (0.0)	0 (0.0)	0 (0.0)	0 (0.0)	0 (0.0)	9 (81.8)	4 (36.4)	0 (0.0)	0 (0.0)	3 (27.3)	0 (0.0)	1 (9.1)	1 (9.1)	2 (18.2)	0 (0.0)	0 (0.0)	2 (18.2)	0 (0.0)	0 (0.0)	0 (0.0)
Total (*n* = 980)	499 (50.9)	466 (47.6)	21 (2.1)	4 (0.4)	8 (0.8)	278 (28.4)	106 (10.8)	21 (2.1)	13 (1.3)	55 (5.6)	34 (3.5)	45 (4.6)	4 (0.4)	165 (16.8)	26 (2.7)	11 (1.1)	85 (8.7)	23 (2.3)	20 (2.0)	38 (3.9)

^*a*^ *P* < 0.05 versus the percentage of PVL-negative MSSA or MRSA strains.

^*b*^ *P* < 0.01 versus the percentage of PVL-negative MSSA or MRSA strains.

^*c*^ *P* < 0.05 versus the percentage of USA300 strains in each classification by Fisher’s exact test.

^*d*^ *P* < 0.01 versus the percentage of USA300 strains in each classification by Fisher’s exact test.

^*e*^ SSTIs, skin and soft tissue infections; ND, no data.

^*f*^ USA300 and ΨUSA300 strains were defined as PVL-positive CC8 MRSA with SCC*mec* types IV and ΨIV, respectively.

### Characterization of the PVL-positive MRSA isolates

**Fig 2:**
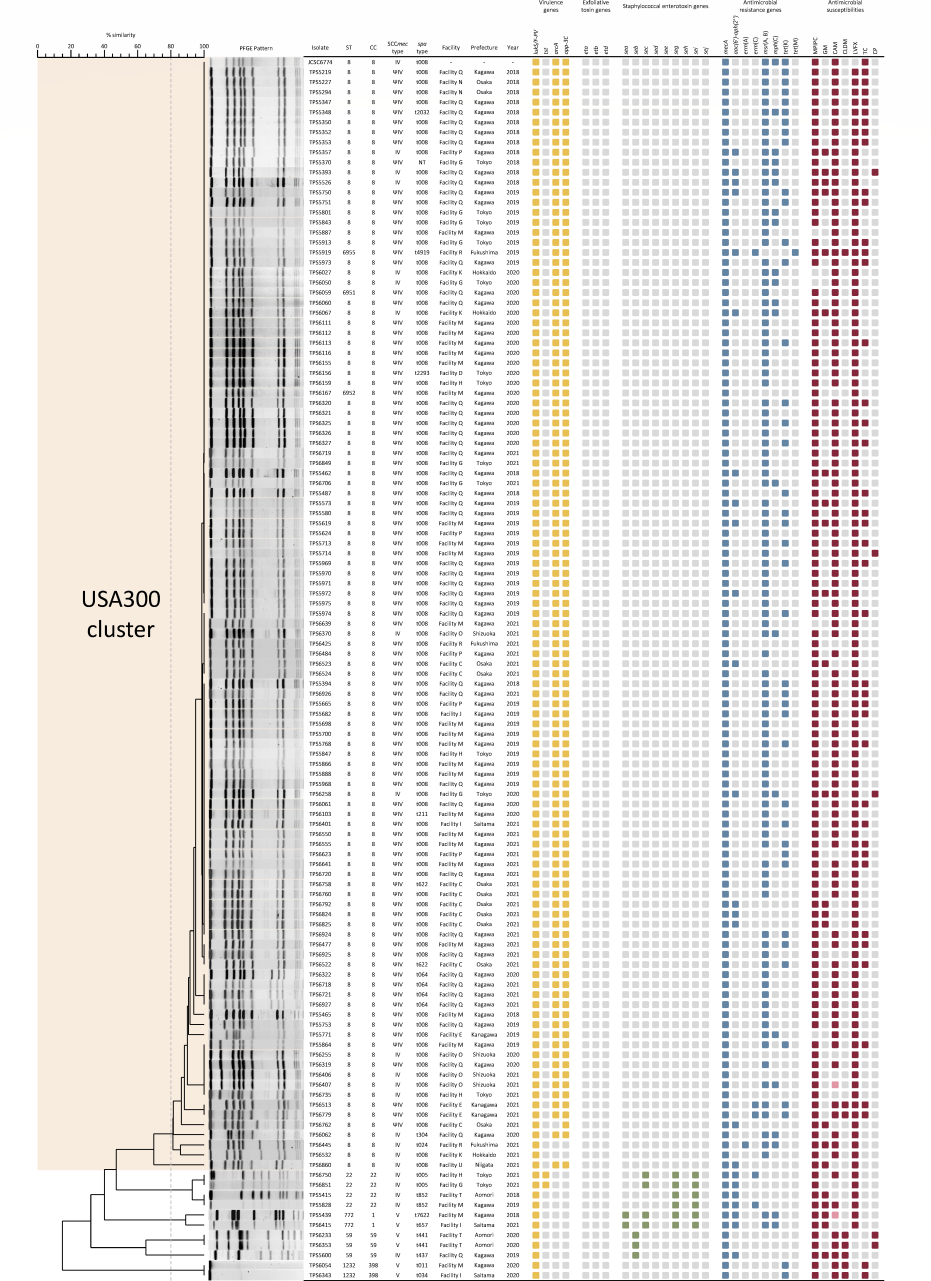
Molecular epidemiological features of PVL-positive MRSA strains isolated in Japan between 2018 and 2021. The presence or absence of genes are indicated by yellow (virulence), green (staphylococcal enterotoxin), blue (antimicrobial resistance), and gray (absence) squares. Antibiotic susceptibility is represented by red (resistant), pink (intermediate), or gray (susceptible) squares. JCSC6774 was used as a reference strain. GM, gentamicin; CAM, clarithromycin; CLDM, clindamycin; CP, chloramphenicol; LVFX, levofloxacin; MPIPC, oxacillin; TC, tetracycline.

PVL-positive MRSA isolates were characterized in detail using *spa* typing, PFGE analysis, and MLST. Among 121 isolates, 110 (90.9%) were classified as clonal complex (CC)8 and formed the USA300 cluster, including the USA300 standard strain (Fig. 2). Although the PFGE pattern of TPS6860 exhibited no more than 80% similarity to the other CC8 strains, it was included in the USA300 cluster for simplicity. The majority of these strains carried *spa* type t008 and ACME type I, which are typical characteristics of USA300. Neither the exfoliative toxin nor staphylococcal enterotoxin genes targeted for detection in this study were found in these strains. There were 11 (9.1%) PVL-positive strains with MLST proﬁles other than CC8, which included four (3.3%) CC22, two (1.7%) CC1, three (2.5%) CC59, and two (1.7%) CC398 strains. The PVL-positive strains had different genes, depending on the MLST profiles. Two CC22 strains carried both PVL and TSST-1 genes. The PFGE patterns of the CC398 strains were not detected because of their resistance to *Sma*I digestion (41).

In this study, of 110 CC8 isolates belonging to the USA300 cluster with SCC*mec* types IV and ΨIV, 16 (14.5%) and 94 (85.5%) were referred to as USA300 and ΨUSA300, respectively (Table 1). The PFGE patterns of USA300 and ΨUSA300 showed 100% similarity in some groups (Fig. 2). ΨUSA300 strains were isolated in Fukushima, Saitama, Tokyo, Kanagawa, Osaka, and Kagawa (Table S3). In contrast, the USA300 strains were isolated from Hokkaido, Fukushima, Tokyo, Niigata, Shizuoka, and Kagawa. ΨUSA300 strains accounted for 13 of 20 (65.0%), 11 of 11 (100.0%), and 68 of 76 (89.5%) of PVL-positive MRSA in the Kanto, Kinki, and Shikoku regions, respectively (Fig. S1A). In the Hokkaido and Chubu regions, all 3 of 3 (100%) and 5 of 5 (100%) PVL-positive MRSA isolates, respectively, were classified as USA300. The proportions of ΨUSA300 and USA300 strains among PVL-positive MRSA did not change significantly from 2018 to 2021, except in 2019 (Fig. S1B). The antimicrobial susceptibilities of USA300 and ΨUSA300 were generally similar, although there were significant differences in the rates of resistance to gentamicin, clarithromycin, and tetracycline (*P* < 0.01, *P* < 0.05, and *P* < 0.01, respectively) (Table 2). CC8 isolates resistant to gentamicin, clarithromycin, and tetracycline tended to carry *aac(6*′*)-aph(2*″), *msr*(A/B), and *tet*(K), respectively (Fig. 2). Comparing the clinical features of these isolates, ΨUSA300 was isolated more frequently than USA300 from patients with deep-seated SSTIs (*P* < 0.05) (Table 1).

**TABLE 2:** Antimicrobial susceptibility of PVL-positive MRSA strains^*c*^ ^*d*^ ^*e*^

Antimicrobial agent	USA300 (*n* = 16)			Ψusa300 (*n* = 94)			Others (*n* = 11)		
	Range	MIC_50_/MIC_90_	No. of resistant strains (%)	Range	MIC_50_/MIC_90_	No. of resistant strains (%)	Range	MIC_50_/MIC_90_	No. of resistant strains (%)
Ampicillin	1–64	16/64	–	0.5–64	16/32	–	1–16	4/16	–
Oxacillin	0.5–64	32/64	14 (87.5)	0.5–64	32/64	91 (96.8)	4–32	16/32	11 (100.0)
Cefalexin	4–128	64/128	–	0.13–≥256	128/128	–	8–128	32/128	–
Cefdinir	1–8	4/8	–	0.25–32	8/16	–	1–8	2/8	–
Faropenem	≤0.06–2	1/2	–	0.13–4	1/2	–	0.13–1	0.25/1	–
Fosfomycin	2–8	2/4	–	2–64	4/8	–	0.13–2	0.5/1	–
Gentamicin	0.13–≥256	0.13/≥256	7 (43.8)	≤0.06–32	0.13/16	11 (11.7)^*b*^	≤0.06–128	4/64	5 (45.5)
Clarithromycin	≤0.06–64	8/64	11 (68.8)	≤0.06–≥256	16/64	85 (90.4)^*a*^	≤0.06–≥256	16/≥256	7 (63.6)
Clindamycin	≤0.06–0.13	≤0.06/0.13	0 (0.0)	≤0.06–≥256	≤0.06/0.13	3 (3.2)	≤0.06–≥256	0.25/≥256	5 (45.5)^*b*^
Levofloxacin	4–32	4/8	16 (100.0)	4–8	4/8	94 (100.0)	0.13–8	4/8	6 (54.5)^*b*^
Nadifloxacin	0.5–2	1/1	–	≤0.06–1	1/1	–	≤0.06–2	1/1	–
Tetracycline	0.13–1	0.13/0.5	0 (0.0)	0.13–128	0.5/64	39 (41.5)^*b*^	0.13–32	0.25/32	2 (18.2)
Minocycline	≤0.06	≤0.06/≤0.06	0 (0.0)	≤0.06–2	≤0.06/0.13	0 (0.0)	≤0.06–0.13	≤0.06/0.13	0 (0.0)
Vancomycin	0.5–1	0.5/1.0	0 (0.0)	0.5–1	1/1	0 (0.0)	0.5–1	0.5/1	0 (0.0)
Chloramphenicol	2–32	4/32	2 (12.5)	2–64	4/8	1 (1.1)	4–32	8/32	2 (18.2)
Fusidic acid	≤0.06–2	≤0.06/0.13	–	≤0.06–0.13	≤0.06/≤0.06	–	≤0.06–0.13	≤0.06/0.13	–

^*a*^ *P* < 0.05 versus the percentage of resistant USA300 strains by Fisher’s exact test.

^*b*^ *P* < 0.01 versus the percentage of resistant USA300 strains by Fisher’s exact test.

^*c*^ MIC_50_/MIC_90_, MIC required to inhibit the growth of 50%/90% of the strains, respectively.

^*d*^ USA300 and ΨUSA300 strains were defined as PVL-positive CC8 MRSA with SCC*mec* types IV and ΨIV, respectively.

^*e*^ –, no breakpoints were defined by CLSI.

## DISCUSSION

This study clarified the current clinical and molecular epidemiology of *S. aureus*, particularly PVL-positive MRSA, among dermatology outpatients in Japan. The proportion of MRSA among *S. aureus* increased gradually from 24.5% to 36.2% during the study period (Fig. 1). The proportions of MRSA among *S. aureus* from patients at the dermatology departments in Japan were reported to be 24.4%, 24.9%, and 25.6% in previous studies and did not vary markedly between 2013 and 2017 (17, 20, 42). Therefore, the prevalence of MRSA in community settings in Japan can be considered to have increased recently, which is a serious problem for achieving a national action plan on antimicrobial resistance based on the World Health Organization Global Action Plan on Antimicrobial Resistance (43). Most MRSA isolates harbored SCC*mec* types IV, ΨIV, and V, typically observed among MRSA isolates in community settings. The results were consistent with those of previous studies (17, 18, 20). The increase in the prevalence of MRSA was most likely due to the dissemination of PVL-positive MRSA because there was no significant change in the prevalence rate of PVL-negative MRSA (Fig. 1). Notably, PVL-positive MRSA accounted for 50.0% of MRSA in 2021, which is substantially higher than that previously reported in Japan (17, 18, 20, 26). The detection rate of PVL genes among MRSA isolates differed significantly by region in Japan (Fig. S1A; Table S3). PVL-positive MRSA is thought to have spread mainly to the Shikoku, Kinki, Chubu, and Kanto regions of Japan. In contrast, the number of PVL-positive MSSA isolates was limited and did not vary significantly by year. The prevalence of PVL-positive *S. aureus* isolates differs greatly among countries (3, 27, 28); the detection rates of PVL genes in this study were higher in MRSA and lower in MSSA compared to other countries. This result might be attributed to the selective pressure on PVL-positive isolates attributed to antibiotic treatment and differences in the frequency of insertion and excision of SCC*mec* by genotype. Further investigation is required to evaluate them. These results are subject to sampling bias because the *S. aureus* isolates used in this study were collected from only 11 of 47 prefectures in Japan, of which 463 of 980 (47.2%) were from Kagawa. However, the prevalence rates of PVL-positive MRSA among MRSA isolates excluding the Shikoku region were 13.8% in 2018, 19.4% in 2019, 34.5% in 2020, and 55.6% in 2021, with a significant increase between 2018 and 2021. Therefore, the prevalence rates of PVL-positive MRSA have possibly increased in regions of Japan other than the Shikoku region.

The presence or absence of *mecA* in *S. aureus* isolates was not associated with the classification of the SSTIs from which they were isolated. Some studies have indicated that MRSA is more virulent than MSSA (44). However, the difference in the risk of MSSA and MRSA infections, especially SSTIs, has not yet been clarified (44, 45). The PVL-negative *S. aureus* were isolated mainly from patients aged ≤14 years with impetigo, whereas the majority of PVL-positive *S. aureus* isolates were from patients aged 15–64 years with deep-seated SSTIs (Table 1; Table S4). These results may reflect the fact that 466 of the 980 (47.6%) *S. aureus* isolates analyzed in this study were obtained from patients with impetigo, which most commonly occurs in children aged 2–5 years (46). PVL-positive isolates can cause deep-seated SSTIs, such as furuncle and cellulitis, mostly in young and middle-aged adults (3, 47); our results are consistent with these reports. The isolation rate of *S. aureu*s in patients with deep-seated SSTIs remained at 49.5% (Table S2), which may be attributed to the difficulty in collecting causative pathogens of these infections or the presence of pathogens other than *S. aureus* causing these infections. Because the samples used in this study were collected as part of routine work, there is a partial lack of patient data and the possible risk of bacterial contamination. More systematic surveillance is needed to accurately assess the clinical background of patients.

In this study, 90.9% of PVL-positive MRSA isolates were classified as USA300 or ΨUSA300, showing similar genetic characteristics (Fig. 2). The USA300 and ΨUSA300 strains isolated in this study were expected to show nearly identical profiles for virulence genes for which the detection process was not conducted, including certain of the genes encoding hemolysin, leukotoxin, staphylococcal enterotoxin, and staphylococcal complement inhibitor, considering the results of previous reports (16, 18). Meanwhile, The MICs and resistance gene proﬁles indicated that USA300 and ΨUSA300 tended to harbor different mobile genetic elements (Table 2). Notably, the number of ΨUSA300 strains carrying SCC*mec* type IV with a particular deletion considerably exceeded the number of USA300 strains. Despite few differences between ΨUSA300 and USA300, previous studies have reported that ΨUSA300 strains formed unique clusters through phylogenetic analysis (16, 18). Therefore, ΨUSA300 strains isolated in this study were expected to show similar results. Since ΨUSA300 strains were isolated in six prefectures belonging to four regions (Table S3), ΨUSA300 could have already been spread to most parts of Japan. To elucidate the reason for the widespread distribution of ΨUSA300, an evaluation of its characteristics using more diverse methods, including comparative genomic analysis, is needed. ΨUSA300 accounted for the majority of PVL-positive MRSA isolated in the Shikoku, Kinki, and Kanto regions (Fig. S1A; Table S3). Meanwhile, only USA300 strains were isolated as PVL-positive MRSA in the Hokkaido and Chubu regions. These results suggest that the major PVL-positive MRSA clones differ by region. ΨUSA300 may have disseminated from the urban areas of Tokyo and Osaka to the surrounding areas and could potentially spread throughout Japan in the future. However, the number of MRSA isolates in the Hokkaido, Chubu, Kinki, and Kyushu regions was small, and no healthcare facilities in the Chugoku region participated in this study. Despite the significant increase in the proportion of PVL-positive MRSA isolates among *S. aureus*, no significant change was recorded in the composition of each clone in PVL-positive MRSA between 2018 and 2021 (Fig. S1B); these results suggest that the prevalence of ΨUSA300, USA300, and other PVL-positive clones may have all increased in the Japanese community. Although the number of USA300 strains was small, ΨUSA300 was more strongly associated with deep-seated SSTIs than USA300 (Table 1). Several USA300 variants share a common ancestor with USA300 (9, 16). ΨUSA300 may have evolved from the same ancestor and subsequently acquired the capability to more frequently cause deep-seated SSTIs than in the USA300. Further studies are necessary to assess the viability and pathogenicity of ΨUSA300, which may exhibit differences compared to that in the USA300 regarding biofilm formation, colonization, invasion, and/or immune evasion. USA300 and its variants have been isolated from countries around Japan, such as Taiwan and South Korea (27, 28); the epidemiology of MRSA in these countries could be related. ΨUSA300 may disseminate to East Asia in the future.

Interestingly, the USA300-LV/J strains, previously isolated in Okinawa, were not identified in this study by detection of COMER and IEC6013 (data not shown) (14). This could be attributed to the lack of ACME, which is related to bacterial adaptability to hosts, in USA300-LV/J (9, 14). In addition to the CC8 strains, PVL genes were detected in the CC22, CC1, CC59, and CC398 strains. Among the four ST22 strains, two were TSST-1-negative, and two were TSST-1-positive. PVL-positive and TSST-1-negative ST22 MRSAs are considered a different lineage from EMRSA-15, the most common ST22 MRSA, and have been isolated in many countries worldwide, including Japan (19, 26, 48). PVL- and TSST-1-positive ST22 MRSA have been sporadically isolated in several countries (48, 49). Recently, this ST22 clone has emerged in several healthcare facilities in Japan and is named ST22-PT (18, 49). The effect of TSST-1 acquisition on the virulence of ST22-PT remains unclear (49). The SCC*mec* type V ST772, SCC*mec* type V ST59, and SCC*mec* type IV ST59 strains were predicted to be the Bengal Bay clone, Taiwan clone, and a sublineage of the Asian-Pacific clone, respectively (50, 51). These three clones have been isolated several times in Japan (17 – 19, 26, 50). The ST1232 strains are considered to be the human-associated clade of CC398 MRSA, of which the livestock-associated clade is widely known (52). This CC398 clone has spread across Southeast Asia and has recently emerged in Japan (41). Although the PVL-positive CC22, CC1, CC59, and CC398 strains exhibited different genotypic and phenotypic proﬁles from the PVL-positive CC8 strains, especially those lacking ACME genes (Fig. 2; Table 2), the experimental data are insufficient to make an accurate comparison of their viability and/or pathogenicity. The prevalence of PVL-positive clones other than USA300 and ΨUSA300 should be carefully monitored.

In conclusion, this study elucidated that the prevalence rate of MRSA in *S. aureus* was elevated in community settings in Japan and that PVL-positive MRSA was responsible for this elevation. ΨUSA300 accounted for most PVL-positive MRSA and mainly caused deep-seated SSTIs. The dissemination of the PVL-positive MRSA clone ΨUSA300 is an emerging threat in Japan, and further studies are needed to elucidate the differences between USA300 and ΨUSA300.

## SUPPLEMENTAL MATERIAL

Fig. S1 spectrum.01248-23-s0001.tif (A) Prevalence rates of PVL-positive MRSA among MRSA isolates in each region of Japan. (B) Annual proportions of PVL-positive MRSA isolates in Japan from 2018 to 2021.

Table S1 spectrum.01248-23-s0002.docx Number of patients (*S. aureus* isolates) included in this study by healthcare facility.

Table S2 spectrum.01248-23-s0003.docx Number of patients (*S. aureus* isolates) included in this study by background of patients.

Table S3 spectrum.01248-23-s0004.docx Number of *S. aureus* isolates from each prefecture.

Table S4 spectrum.01248-23-s0005.docx Background of patients from whom *S. aureus* strains belonging to each genotype were isolated.

ASM does not own the copyrights to Supplemental Material that may be linked to, or accessed through, an article. The authors have granted ASM a non-exclusive, world-wide license to publish the Supplemental Material files. Please contact the corresponding author directly for reuse.
