# Supplementary figures and images for: Significant increase in the prevalence of Panton–Valentine leukocidin-positive methicillin-resistant Staphylococcus aureus, particularly the USA300 variant ΨUSA300, in the Japanese community
Source: Microbiol Spectr. 2023 Nov 6;11(6):e01248-23. doi: 10.1128/spectrum.01248-23 (PMC10715091; doi:10.1128/spectrum.01248-23)

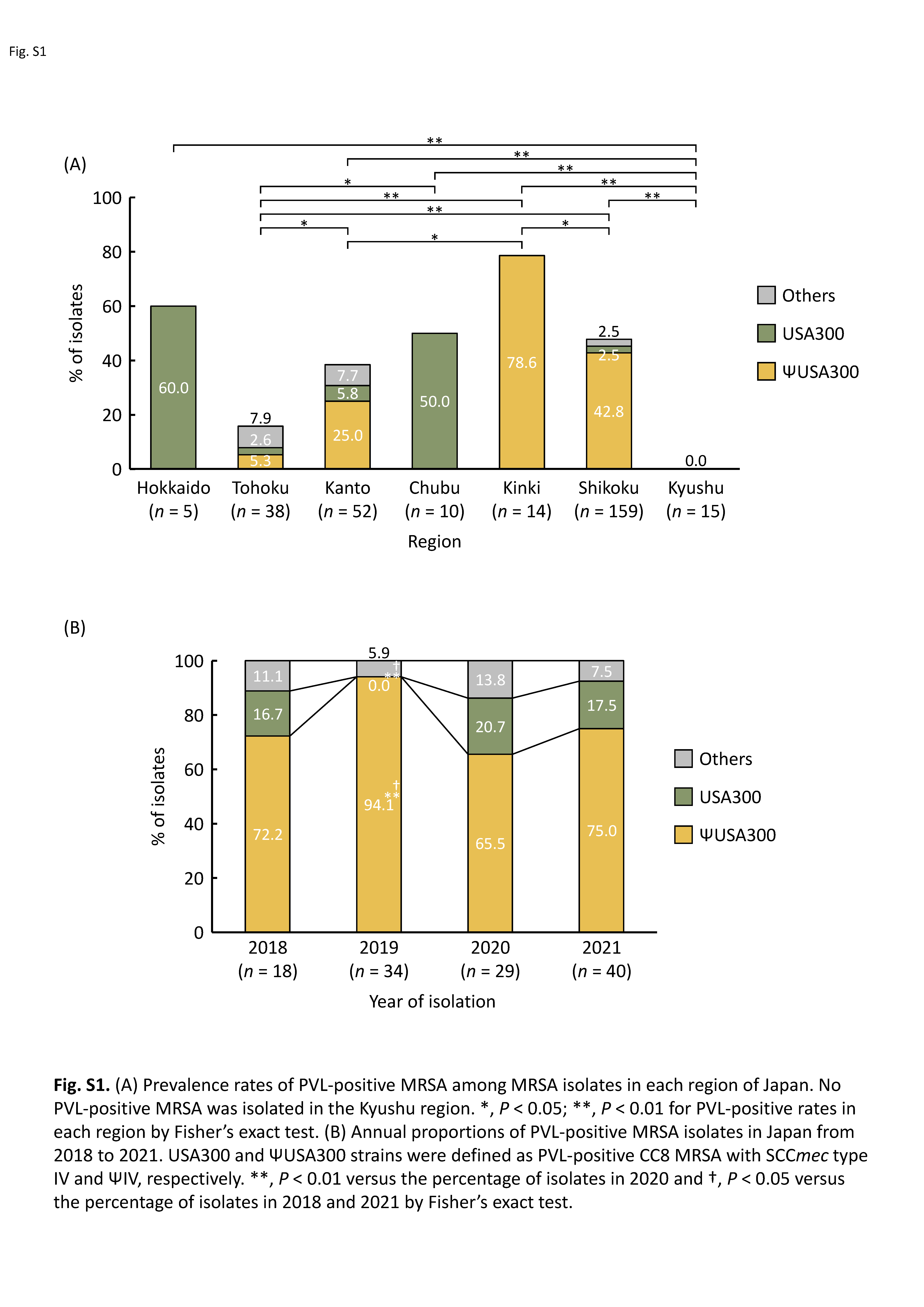

Supplement: Fig. S1 — (A) Prevalence rates of PVL-positive MRSA among MRSA isolates in each region of Japan. (B) Annual proportions of PVL-positive MRSA isolates in Japan from 2018 to 2021. [file spectrum.01248-23-s0001.tif]
