# Supplementary material for: Significant increase in the prevalence of Panton–Valentine leukocidin-positive methicillin-resistant Staphylococcus aureus, particularly the USA300 variant ΨUSA300, in the Japanese community
Source: Microbiol Spectr. 2023 Nov 6;11(6):e01248-23. doi: 10.1128/spectrum.01248-23 (PMC10715091; doi:10.1128/spectrum.01248-23)
Supplement: Table S1 — Number of patients (S. aureus isolates) included in this study by healthcare facility. [file spectrum.01248-23-s0002.docx]

**Table S1.** Number of patients (*S. aureus* isolates) included in this study by healthcare facility

| Healthcare facility | Prefecture | Year of isolation | | | | Total |
| --- | --- | --- | --- | --- | --- | --- |
|  |  | 2018 | 2019 | 2020 | 2021 |  |
| Clinic A | Hokkaido | 30 (7) | 20 (5) | 20 (4) | 19 (6) | 89 (22) |
| Clinic B | Aomori | 52 (18) | 49 (10) | 123 (40)* | 87 (28) | 311 (96) |
| Clinic C | Fukushima | 0 (0) | 60 (35) | 40 (27) | 20 (6) | 120 (68) |
| Clinic D | Fukushima | 0 (0) | 6 (5) | 0 (0) | 0 (0) | 6 (5) |
| Hospital A | Saitama | 0 (0) | 0 (0) | 14 (3) | 17 (10) | 31 (13) |
| Clinic E | Tokyo | 25 (24)* | 18 (17) | 14 (13) | 17 (16) | 74 (70) |
| Clinic F | Tokyo | 9 (6) | 28 (13) | 12 (7) | 19 (9) | 68 (35) |
| Clinic G | Tokyo | 30 (28) | 10 (10) | 15 (15) | 0 (0) | 55 (53) |
| Clinic H | Tokyo | 0 (0) | 18 (9) | 4 (0) | 0 (0) | 22 (9) |
| Clinic I | Tokyo | 1 (1) | 7 (6) | 6 (5) | 3 (3) | 17 (15) |
| Hospital B | Kanagawa | 6 (3) | 20 (10) | 14 (4) | 16 (6) | 56 (23) |
| Clinic J | Niigata | 0 (0) | 0 (0) | 0 (0) | 1 (1) | 1 (1) |
| Hospital C | Shizuoka | 10 (6) | 3 (0) | 16 (7) | 23 (14) | 52 (27) |
| Clinic K | Osaka | 30 (15) | 20 (14) | 0 (0) | 0 (0) | 50 (29) |
| Clinic L | Osaka | 0 (0) | 0 (0) | 0 (0) | 19 (13) | 19 (13) |
| Clinic M | Kagawa | 30 (15) | 61 (39) | 40 (22)** | 56 (27) | 187 (103) |
| Hospital D | Kagawa | 59 (57)* | 60 (59) | 20 (20) | 22 (22) | 161 (158) |
| Clinic N | Kagawa | 30 (19) | 20 (8) | 20 (10) | 20 (18)** | 90 (55) |
| Clinic O | Kagawa | 30 (26) | 26 (23) | 11 (10) | 20 (20)* | 87 (79) |
| Clinic P | Kagawa | 24 (23) | 22 (20) | 13 (10) | 12 (12) | 71 (65) |
| Clinic Q | Kagawa | 3 (1) | 4 (2) | 0 (0) | 0 (0) | 7 (3) |
| Clinic R | Kumamoto | 12 (8) | 15 (12) | 8 (8) | 10 (10) | 45 (38) |
| Total | | 381 (257) | 467 (297) | 390 (205) | 381 (221) | 1619 (980) |

* and ** indicate that both MSSA and MRSA were isolated from one and two patients, respectively.
