# Supplementary material for: Significant increase in the prevalence of Panton–Valentine leukocidin-positive methicillin-resistant Staphylococcus aureus, particularly the USA300 variant ΨUSA300, in the Japanese community
Source: Microbiol Spectr. 2023 Nov 6;11(6):e01248-23. doi: 10.1128/spectrum.01248-23 (PMC10715091; doi:10.1128/spectrum.01248-23)
Supplement: Table S2 — Number of patients (S. aureus isolates) included in this study by background of patients. [file spectrum.01248-23-s0003.docx]

**Table S2.** Number of patients (*S. aureus* isolates) included in this study by background of patients

| Background of patients | | Superﬁcial SSTIs | | | | | Deep-seated SSTIs | | | | | | | | Unclassifiable | | | | | | ND | Total |
| --- | --- | --- | --- | --- | --- | --- | --- | --- | --- | --- | --- | --- | --- | --- | --- | --- | --- | --- | --- | --- | --- | --- |
|  |  |  | Impetigo | Folliculitis | Pustule | Others |  | Furuncle | Furunculosis | Carbuncle | Abscess | Cellulitis | Paronychia | Others |  | Epidermal cyst | Hidradenitis suppurativa | Ulcer | Wound | Others |  |  |
| Age (years) | <14 | 490 (440) | 482 (436)*** | 7 (3) | 0 (0) | 1 (1) | 85 (49) | 49 (25) | 9 (9) | 1 (0) | 9 (6) | 0 (0) | 16 (9) | 1 (0) | 24 (9) | 12 (2) | 2 (0) | 2 (1) | 6 (6)* | 2 (0) | 11 (7) | 610 (505) |
|  | 15–64 | 86 (39) | 25 (20) | 37 (11) | 5 (4) | 19 (4) | 298 (136) | 111 (47) | 9 (9) | 14 (10) | 78 (33)* | 38 (19) | 47 (17) | 1 (1) | 194 (74) | 98 (18) | 16 (9) | 50 (32) | 15 (7) | 15 (8) | 13 (7) | 591 (256) |
|  | >65 | 40 (15) | 17 (8) | 16 (4) | 2 (0) | 5 (3) | 151 (65) | 85 (28)* | 1 (1) | 1 (1) | 25 (9) | 14 (10) | 22 (13) | 3 (3) | 121 (45) | 32 (4) | 1 (0) | 64 (33)* | 10 (3) | 14 (5) | 15 (9) | 327 (134) |
|  | ND | 6 (5) | 2 (2) | 4 (3) | 0 (0) | 0 (0) | 28 (28) | 6 (6) | 2 (2) | 2 (2) | 7 (7) | 5 (5) | 6 (6) | 0 (0) | 38 (37) | 2 (2) | 2 (2) | 19 (19) | 7 (7) | 8 (7) | 19 (15) | 91 (85) |
| Sex | Male | 358 (287) | 302 (268)** | 40 (13) | 3 (1) | 13 (5) | 320 (159) | 147 (63)* | 12 (12) | 8 (5) | 76 (33)* | 40 (24) | 36 (21) | 1 (1) | 228 (97) | 89 (14) | 17 (9) | 77 (47) | 23 (14)* | 22 (13) | 25 (15) | 931 (558) |
|  | Female | 257 (206) | 218 (192)** | 23 (8) | 4 (3) | 12 (3) | 240 (117) | 104 (43) | 9 (9) | 10 (8) | 41 (20) | 17 (10) | 55 (24) | 4 (3) | 146 (65) | 55 (12) | 3 (1) | 56 (36)* | 15 (9) | 17 (7) | 17 (10) | 660 (398) |
|  | ND | 7 (6) | 6 (6) | 1 (0) | 0 (0) | 0 (0) | 2 (2) | 0 (0) | 0 (0) | 0 (0) | 2 (2) | 0 (0) | 0 (0) | 0 (0) | 3 (3) | 0 (0) | 1 (1) | 2 (2) | 0 (0) | 0 (0) | 16 (13) | 28 (24) |
| Total | | 622 (499) | 526 (466) | 64 (21) | 7 (4) | 25 (8) | 562 (278) | 251 (106) | 21 (21) | 18 (13) | 119 (55) | 57 (34) | 91 (45) | 5 (4) | 377 (165) | 144 (26) | 21 (11) | 135 (85) | 38 (23) | 39 (20) | 58 (38) | 1619 (980) |

SSTIs, skin and soft tissue infections; ND, no data.

*, **, and *** indicate that both MSSA and MRSA were isolated from one, two and four patients, respectively.
