# Supplementary material for: Significant increase in the prevalence of Panton–Valentine leukocidin-positive methicillin-resistant Staphylococcus aureus, particularly the USA300 variant ΨUSA300, in the Japanese community
Source: Microbiol Spectr. 2023 Nov 6;11(6):e01248-23. doi: 10.1128/spectrum.01248-23 (PMC10715091; doi:10.1128/spectrum.01248-23)
Supplement: Table S3 — Number of S. aureus isolates from each prefecture. [file spectrum.01248-23-s0004.docx]

**Table S3.** Number of *S. aureus* isolates from each prefecture

| Region | Prefecture | *S. aurues* | PVL-negative MSSA | PVL-positive MSSA | PVL-negative MRSA | PVL-positive MRSA | | | |
| --- | --- | --- | --- | --- | --- | --- | --- | --- | --- |
|  |  |  |  |  |  |  | USA300 | ΨUSA300 | Others |
| Hokkaido | Hokkaido | 22 | 15 | 2 | 2 | 3 | 3 | 0 | 0 |
| Tohoku | Aomori | 96 | 74 | 0 | 19 | 3 | 0 | 0 | 3 |
|  | Fukushima | 73 | 57 | 0 | 13 | 3 | 1 | 2 | 0 |
| Kanto | Saitama | 13 | 6 | 0 | 4 | 3 | 0 | 1 | 2 |
|  | Tokyo | 182 | 140 | 3 | 25 | 14 | 3 | 9 | 2 |
|  | Kanagawa | 23 | 15 | 2 | 3 | 3 | 0 | 3 | 0 |
| Chubu | Niigata | 1 | 0 | 0 | 0 | 1 | 1 | 0 | 0 |
|  | Shizuoka | 27 | 15 | 3 | 5 | 4 | 4 | 0 | 0 |
| Kinki | Osaka | 42 | 25 | 3 | 3 | 11 | 0 | 11 | 0 |
| Shikoku | Kagawa | 463 | 301 | 3 | 83 | 76 | 4 | 68 | 4 |
| Kyushu | Kumamoto | 38 | 23 | 0 | 15 | 0 | 0 | 0 | 0 |
| Total | | 980 | 671 | 16 | 172 | 121 | 16 | 94 | 11 |
