# Supplementary material for: Significant increase in the prevalence of Panton–Valentine leukocidin-positive methicillin-resistant Staphylococcus aureus, particularly the USA300 variant ΨUSA300, in the Japanese community
Source: Microbiol Spectr. 2023 Nov 6;11(6):e01248-23. doi: 10.1128/spectrum.01248-23 (PMC10715091; doi:10.1128/spectrum.01248-23)
Supplement: Table S4 — Background of patients from whom S. aureus strains belonging to each genotype were isolated. [file spectrum.01248-23-s0005.docx]

**Table S4.** Background of patients from whom *S. aureus* strains belonging to each genotype were isolated

| Genotype of *S. aureus* strains | | No. of strains (%) by age (years) | | | | Mean of age (years) | Median of age (years) | Range of age (years) | No. of strains (%) by sex | | | male-to-female sex ratio |
| --- | --- | --- | --- | --- | --- | --- | --- | --- | --- | --- | --- | --- |
|  |  | <14 | 15–64 | >65 | ND |  |  |  | Male | Female | ND |  |
| PVL-negative MSSA (*n* = 671) | | 375 (55.9) | 148 (22.1) | 93 (13.9) | 55 (8.2) | 24.0 | 8 | 0–98 | 381 (56.8) | 273 (40.7) | 17 (2.5) | 1.40 |
| PVL-positive MSSA (*n* = 16) | | 3** (18.8) | 12** (75.0) | 0 (0.0) | 1 (6.3) | 33.0‡ | 30 | 3–57 | 10 (62.5) | 6 (37.5) | 0 (0.0) | 1.67 |
| PVL-negative MRSA (*n* = 172) | | 100 (58.1) | 28 (16.3) | 28 (16.3) | 16 (9.3) | 24.6 | 7 | 0–92 | 96 (55.8) | 71 (41.3) | 5 (2.9) | 1.35 |
| PVL-positive MRSA (*n* = 121) | | 27** (22.3) | 68** (56.2) | 13 (10.7) | 13 (10.7) | 29.7‡‡ | 25 | 0–90 | 71 (58.7) | 48 (39.7) | 2 (1.7) | 1.48 |
|  | USA300 (*n* = 16) | 5 (31.3) | 6 (37.5) | 4 (25.0) | 1 (6.3) | 33.7 | 24 | 1–86 | 8 (50.0) | 8 (50.0) | 0 (0.0) | 1.00 |
|  | ΨUSA300 (*n* = 94) | 20 (21.3) | 57 (60.6) | 6† (6.4) | 11 (11.7) | 27.3 | 24 | 0–79 | 55 (58.5) | 37 (39.4) | 2 (2.1) | 1.49 |
|  | Others (*n* = 11) | 2 (18.2) | 5 (45.5) | 3 (27.3) | 1 (9.1) | 43.0 | 32 | 8–90 | 8 (72.7) | 3 (27.3) | 0 (0.0) | 2.67 |
| Total (*n* = 972) | | 500 (51.4) | 255 (26.2) | 132 (13.6) | 85 (8.7) | 24.9 | 9 | 0–98 | 553 (56.9) | 395 (40.6) | 24 (2.5) | 1.40 |

USA300 and ΨUSA300 strains were defined as PVL-positive CC8 MRSA with SCC*mec* type IV and ΨIV, respectively.

**, *P* < 0.01 versus the percentage of PVL-negative MSSA or MRSA strains and †, *P* < 0.05 versus the percentage of USA300 strains in each classification by Fisher’s exact test.

‡, *P* < 0.05; ‡‡, *P* < 0.01 versus the age distribution of patients with PVL-negative MSSA or MRSA strains by Mann–Whitney U test.

ND, no data.
